# Dance Training Improves Cytokine Secretion and Viability of Neutrophils in Diabetic Patients

**DOI:** 10.1155/2019/2924818

**Published:** 2019-11-20

**Authors:** Leandro Borges, Maria E. P. Passos, Maysa B. B. Silva, Vinicius C. Santos, César M. Momesso, Tania C. Pithon-Curi, Renata Gorjão, Stuart R. Gray, Kauê C. A. Lima, Paulo B. de Freitas, Elaine Hatanaka

**Affiliations:** ^1^Institute of Physical Activity and Sports Sciences, Cruzeiro do Sul University, São Paulo 01506-000, Brazil; ^2^São Paulo University (USP), São Paulo 05508-900, Brazil; ^3^Institute of Cardiovascular & Medical Sciences, University of Glasgow, Glasgow G12 8TA, UK; ^4^Department of Neurology, Milton S. Hershey Medical Center, The Pennsylvania State University, Hershey, PA 16801, USA; ^5^Department of Kinesiology, The Pennsylvania State University, University Park, PA 16802, USA

## Abstract

**Background:**

Evidence suggests that exercise improves neutrophil function. The decreased functional longevity of neutrophils and their increased clearance from infectious sites contribute to the increased susceptibility to infection and severity of infection observed in patients with diabetes.

**Objective:**

Herein, we investigated the effects of a dance program on neutrophil number, function, and death in type 2 diabetes mellitus (T2DM) patients and healthy volunteers.

**Methods:**

Ten patients with T2DM and twelve healthy individuals participated in a moderate-intensity dance training program for 4 months. The plasma levels of leptin, free fatty acids (FFAs), tumour necrosis factor-*α* (TNF-*α*), C-reactive protein (CRP), interleukin-1*β* (IL-1*β*), and interleukin-1 receptor antagonist (IL-1ra); neutrophil counts; extent of DNA fragmentation; cell membrane integrity; and production of TNF-*α*, interleukin-8 (IL-8), interleukin-6 (IL-6), and IL-1*β* in neutrophils were measured before and after training.

**Results:**

Training reduced plasma levels of TNF-*α* (1.9-fold in controls and 2.2-fold in patients with T2DM) and CRP (1.4-fold in controls and 3.4-fold in patients with T2DM). IL-1ra levels were higher in the control group (2.2-fold) after training. After training, neutrophil DNA fragmentation was decreased in patients with T2DM (90%), while the number of neutrophils increased (70% in controls and 1.1-fold in patients with T2DM).

**Conclusion:**

Dance training is a nonpharmacological strategy to reduce inflammation and improve neutrophil clearance in patients with T2DM.

## 1. Introduction

Neutrophil apoptosis, an important component of inflammation, regulates neutrophil functional longevity and resolution. Evidence suggests that defects in the apoptosis of neutrophils contribute to increased infection and mortality rates in patients with diabetes [1]. In fact, elevated levels of inflammatory markers [interleukin-1*β* (IL-1*β*), tumour necrosis factor-*α* (TNF-*α*), interleukin-6 (IL-6), interleukin-8 (IL-8), and C-reactive protein (CRP)] were shown to increase neutrophil apoptosis and impair neutrophil clearance in patients with diabetes [2]. This dysfunction leads to the inadequate activation of leukocytes, advances the progression of diabetic complications, and increases the risk of invasive microorganisms [3–5].

Dancing is indicated for the management of diabetes to improve psychosocial parameters, motor control, insulin sensitivity, vascular health, and the efficiency of immune function [6]. The beneficial effects of exercise are mediated partly by control over the serum levels of inflammatory mediators [7]. However, the effects of moderate-intensity dancing on neutrophil viability, the apoptosis ratio, and function in exercise and diabetes have not been reported.

Herein, we studied the effects of a 4-month dance training program (60 min/day, twice a week) on body composition and the plasma levels of FFAs, leptin, CRP, IL-1*β*, TNF-*α*, and interleukin-1 receptor antagonist (IL-1ra). Additionally, we evaluated the proportions of necrotic and apoptotic neutrophils and the release of cytokines (IL-8, TNF-*α*, IL-1*β*, and IL-6) by neutrophils under basal conditions and following lipopolysaccharide (LPS) stimulation.

## 2. Methods

### 2.1. Subjects

One hundred women were enrolled, forty volunteers started the study intervention, and 22 participants completed the study intervention. Ten volunteers with T2DM and twelve healthy controls with no previous experience with Zumba or Brazilian folkloric dances participated in this research. In order to strengthen adherence and permanence to the dance program, this study chose to focus on the inclusion of women. The main reasons for dropping out were financial, health, and family problems. Female volunteers with T2DM diagnosed according to the criteria established by the American Diabetes Association [8] at least 10 years earlier were recruited from a medical centre specializing in diabetes (Associação Nacional de Assistência ao Diabético (ANAD)). Experimental procedures were conducted according to the Declaration of Helsinki after approval from the Ethics Committee of the Cruzeiro do Sul University (CE/UCS-018/2014). Participants with inflammatory diseases and those who were taking anti-inflammatory drugs were excluded from the research.

Participants' body mass indices (BMIs) were calculated [BMI = weight [kilogram/height^2^ (m^2^)], and their waist circumferences at the midpoint between the lower border of the rib cage and the iliac crest were measured with a nonstretchable metric tape. Body fat was determined using a tetrapolar bioimpedance device (Biodynamics Corporation, 310, EUA). According to BMI, participants were considered overweight and baseline patient characteristics are reported in Table 1.

Physical activity levels (type of physical activity and number of hours per week) were assessed by the short International Physical Activity Questionnaire (IPAQ) and a pedometer worn for 7 consecutive days (Table 1). Regarding the IPAQ questionnaire, most participants reported participating in daily walks and this was consistent with the pedometer step count data. According to normative categories of steps/day by sex and age group, data involving pedometer step counts were between “average” and “above average” [9].

Blood pressure (BP) was measured three times with a mercury sphygmomanometer, and blood glucose was analysed by an automatic analyser (Accu-Chek Active, Roche, São Paulo (SP)) (Table 1). The use of specific medications for diabetes and/or other diseases was determined with an open-ended question (see [Supplementary-material supplementary-material-1] in the Supplementary Material).

### 2.2. Exercise Intervention

Dance training consisted of 60 min of exercise carried out twice a week for 4 months. Each session consisted of the following exercises: a 5 min warm-up, 25 min of Zumba dance, 25 min of Brazilian folkloric dances (*forró* and *samba*), and 5 min of stretching exercises.

The dance program was conducted in São Paulo (Brazil), and all classes were performed in the afternoon, between 4pm to 6pm, with a rest period of 24-48 hours from the previous class. For the convenience of the participants, volunteers with T2DM had their training in a medical centre for people with diabetes (ANAD) while healthy participants had dance training at the Cruzeiro do Sul University. Regardless of location, both groups had the same training conditions: all classes were taught by the same certified instructor using the same dance protocol in a spacious room with appropriate facilities for a dance class (chairs for resting, stereo with moderate volume, and floor without deformities).

To assess the intensity of the dance, each participant was monitored with a Polar FT7M heart rate (HR) monitor during the training period. We recorded the average resting HR (HR rest) and the average HR during the dance class (HR during class). Participants were asked to sit for 15 minutes, before class, to obtain the resting HR. The maximum HR was collected during a routine dance class, using a wireless heart rate monitor with a watch-like receiver worn on the wrist to compute the data of each participant. After calculating the HR reserve, the training intensity was classified as moderate according to the guidelines of the American College of Sports Medicine [10]. Intensity calculations revealed the following percentage HR reserve values: 58.1 ± 1.7% (control group), 58.4 ± 1.8% (control group after training), 57.2 ± 3.1% (T2DM group), and 56.4 ± 2.5% (T2DM group after training).

### 2.3. Sample Collection and Cell Purification

Prior to blood sampling, participants fasted for 12 h and were asked to avoid any exercise for the preceding 72 h period. Before and 4 months after dance training, a total of 20 millilitres of blood was drawn into two BD vacutainer tubes containing heparin, centrifuged at 400 × *g* for 10 min and subjected to cell separation and plasma collection. Plasma was stored at −80°C prior to cytokine, adipokine, fructosamine, and lipoprotein analyses. Human neutrophils were separated by Histopaque 1077 (Sigma Chemical Co., St. Louis, MO) to an endotoxin-free state following the supplier's instructions and as previously described by Böyum [11].

After isolation, neutrophils (2.5 × 10^6^) were suspended in Roswell Park Memorial Institute (RPMI) 1640 medium supplemented with 0.3 g/L glutamine, 2.32 g/L Hepes, 2 g/L sodium bicarbonate, 100 *μ*g/mL streptomycin, 100 UI/mL penicillin, and 10% fetal bovine serum. The cells were then counted in a Neubauer chamber and immediately cultured at 37°C and 5% CO_2_, with and without LPS (5 *μ*g/mL) supplied by Sigma Chemical Co. (St. Louis, MO). After 4 hours of cell culture, the supernatant was collected and stored at ≤−80°C prior to cytokine analysis.

### 2.4. Determination of Fructosamine Content

The fructosamine content was measured by spectrometry according to a protocol described by Johnson et al. [12] and the supplier's instructions (Labtest, Minas Gerais, Brazil).

### 2.5. Determination of the FFA Concentration

The FFA concentration was assessed by the enzymatic, colorimetric method described by Duncombe [13]. Kits to detect FFAs were provided by Wako Diagnostics (Mountain View, USA). FFA concentrations were determined by measuring the absorbance at 550 nm, and the lower limit of detection for the FFA analysis was 0.016 mEq/L.

### 2.6. Determination of Lipoprotein Levels

Lipoproteins were measured by spectrometry. High-density lipoprotein (HDL), triglycerides (TG), and cholesterol detection kits (Roche Diagnostics, Indianapolis, USA) were used according to the supplier's instructions. The plasma low-density lipoprotein (LDL) concentration was calculated using the Friedewald formula [14].

### 2.7. Determination of Inflammatory Markers

Using DuoSet ELISA kits (R&D Systems, Minneapolis, MN, USA), IL-6, IL-1*β*, TNF-*α*, IL-1ra, CRP, IL-8, and leptin levels were analysed following the supplier's instructions and as described previously [15, 16]. The intra-assay and interassay coefficients of variance were 3–5% and 8–10%, respectively.

### 2.8. Cell Viability Assay

After their isolation and centrifugation at 400 × *g* for 15 min, neutrophils (1 × 10^6^ cells) were suspended in 500 *μ*L PBS (pH 7.4). Thereafter, 50 microlitres (*μ*L) of a propidium iodide (PI) solution [50 milligrams per millilitre (mg/mL) in PBS] was added to the cell mixture. With a FACSCalibur flow cytometer (Becton Dickinson Systems, CA, USA), the viability of the neutrophils was analysed. Neutrophil viability was determined using PI fluorochrome (0.05% in PBS). A total of 10,000 events per sample were analysed. The fluorescence intensity was assessed by FL2 channels (orange-red fluorescence = 585/545 nm) [17, 18].

### 2.9. DNA Fragmentation

Through flow cytometry (Sigma Chemical Co., St. Louis, MO), DNA fragmentation immediately after DNA staining with PI was analysed. Fluorescence was determined using the FL2 channel, and 10,000 events per sample were assessed (orange-red fluorescence = 585/545 nm) according to a protocol previously described by Nicoletti et al. [18] and Cury-Boaventura et al. [19].

### 2.10. Statistical Analysis

For blood analysis, outliers were removed from normally distributed data in accordance with the criteria of Chauvenet [20], in which values higher or lower than two standard deviations in each group were removed. Therefore, the results of blood analysis are expressed in duplicate as the mean ± standard error (SE) of at least seven participants per group. Analyses were performed by comparing the following groups: control before training, control after training, participants with T2DM before training, and participants with T2DM after training. Analyses of anthropometric values were performed by Student's *t*-test, and statistical analyses of differences between groups and time periods (analyses of cytokine levels, plasma CRP levels, the neutrophil count, DNA fragmentation, and membrane cell integrity) were performed by two-way mixed ANOVA with Bonferroni correction for multiple comparisons by GraphPad Prism software (INStat; GraphPad Software, San Diego, CA, USA). The significance level was set to *p* < 0.05.

## 3. Results

**Figure 1:**
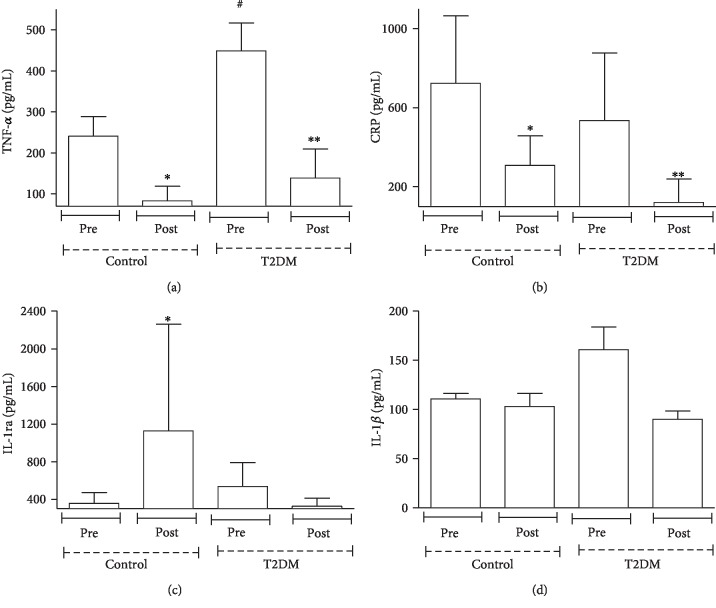
Effects of dance training on the plasma concentrations of TNF-*α*, CRP, IL-1ra, and IL-1*β*. Values in participants with T2DM and healthy participants were quantified before and four months after dance training. Data are represented as the mean ± SE of at least 7 participants per group. ^∗^*p* < 0.01 precontrol group vs. postcontrol group; ^∗∗^*p* < 0.01 pre-T2DM group vs. post-T2DM group; ^#^*p* < 0.01 precontrol group vs. pre-T2DM group.

Our study noted that basal TNF-*α* levels were higher (90%) (Figure 1(a)) in patients with T2DM than in the control group. After dance training, a reduction in the plasma levels of TNF-*α* (1.9-fold in controls and 2.2-fold in patients with T2DM) (Figure 1(a)) and CRP (1.4-fold in controls and 3.4-fold in patients with T2DM) (Figure 1(b)) was observed. In concordance with the anti-inflammatory effect of dance practice, the plasma level of IL-1ra (Figure 1(c)) in the control group increased (2.2-fold) after the exercise period. However, dance training did not modify plasma IL-1*β* (Figure 1(d)) or body composition, fructosamine levels, plasma leptin levels, plasma glucose levels, and plasma FFA levels in the control group and patients with T2DM (Table 2).

**Figure 2:**
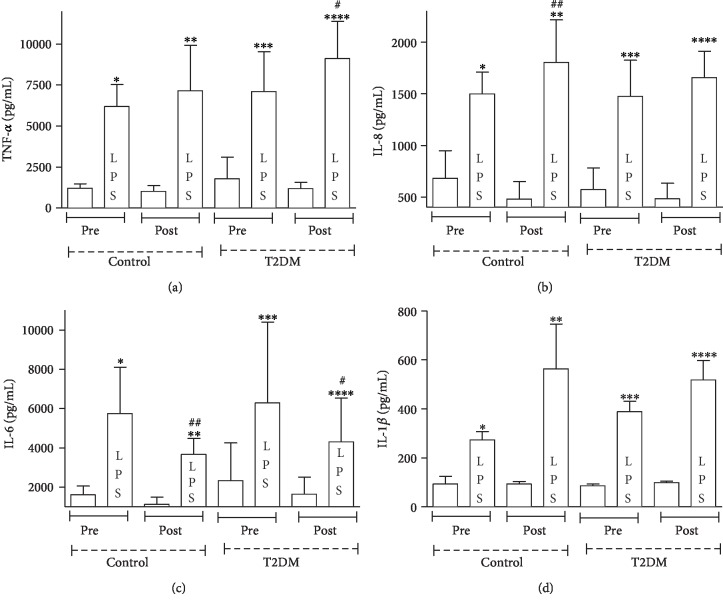
Effects of dance training on the levels of TNF-*α*, IL-8, IL-6, and IL-1*β* produced by neutrophils determined under basal and LPS-stimulated conditions. Values in participants with T2DM and healthy participants were quantified before and four months after dance training. Data are represented as the mean ± SE of at least 7 participants per group. ^∗^*p* < 0.05 precontrol vs. precontrol (LPS); ^∗∗^*p* < 0.05 postcontrol vs. postcontrol (LPS); ^∗∗∗^*p* < 0.05 pre-T2DM vs. pre-T2DM (LPS); ^∗∗∗∗^*p* < 0.05 post-T2DM vs. post-T2DM (LPS); ^#^*p* < 0.05 pre-T2DM (LPS) vs. post-T2DM (LPS); ^##^*p* < 0.05 precontrol (LPS) vs. postcontrol (LPS).

Initially, Figure 2 shows that, at baseline, there was no difference for neutrophil TNF-*α*, IL-8, IL-6, and IL-1*β* production when compared participants in the control group and participants with T2DM before training (*p* > 0.05). Nevertheless, after training, there was an increase in neutrophil TNF-*α* (30% in patients with T2DM) (Figure 2(a)) and IL-8 (20% in controls) (Figure 2(b)) production, and neutrophil IL-6 production was lower (60% in controls and 50% in patients with T2DM) in both groups following LPS stimulation (Figure 2(c)). There was no difference in neutrophil IL-1*β* production between the groups (*p* > 0.05) (Figure 2(d)).

Comparing the T2DM group before and after training, we observed that LPS-stimulated neutrophil TNF-*α* production was higher in the posttraining period (Figure 2(a)). Similarly, the same pattern is observed in the control group when comparing the moments before and after training, with increased IL-8 production of LPS-stimulated neutrophils after training (Figure 2(b)).

**Figure 3:**
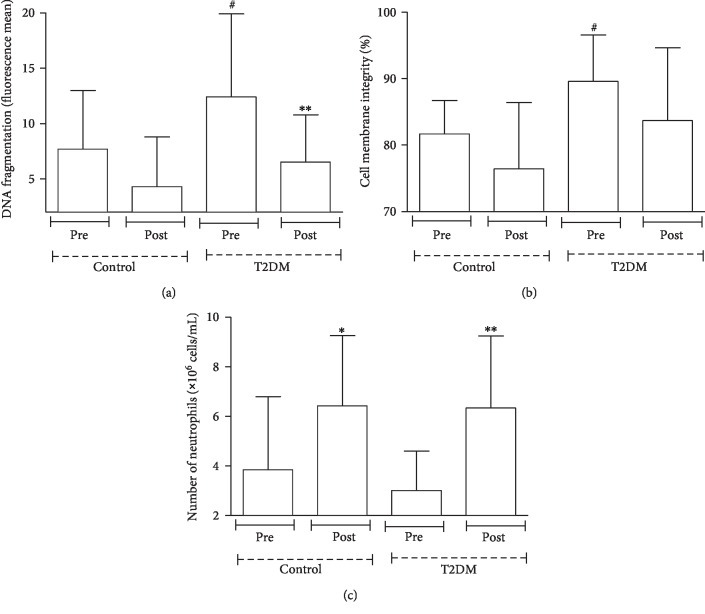
Number of neutrophils, the extent of DNA fragmentation, and membrane cell integrity in participants with T2DM and healthy participants before and four months after dance training. Data are represented as the mean ± SE of at least 7 participants per group. ^∗^*p* < 0.05 precontrol group vs. postcontrol group; ^∗∗^*p* < 0.05 pre-T2DM group vs. post-T2DM group; ^#^*p* < 0.05 precontrol group vs. pre-T2DM group.

Under basal conditions, there were 60% more neutrophils with fragmented DNA in patients with T2DM than in the control group (Figure 3(a)). Figure 3(a) also shows a reduction in the number of neutrophils with fragmented DNA in patients with T2DM after dance training (90%). After training, there was no difference in the analysis of cell membrane integrity (Figure 3(b)), but there was an elevation in the number of neutrophils (70% in controls and 1.1-fold in patients with T2DM) (Figure 3(c)).

## 4. Discussion

Our results show that people with T2DM and healthy participants reduced plasma inflammatory markers and acute-phase proteins, as well as an increased concentration of anti-inflammatory cytokines after a dance program. These alterations were associated with altered cytokine production by neutrophils in LPS stimulus and a reduction in neutrophil death. To our knowledge, this research was the first to investigate neutrophil function and death after a moderate-intensity dance program in people with T2DM.

Our study noted a decrease in plasma TNF-*α* and CRP levels and an increase in IL-1ra levels in both controls and patients with T2DM after dance training, and these effects occurred without changes in body composition. Although changes in body composition are important in some physiological and inflammatory events, there is growing evidence that exercise, even in the absence of weight loss, directly influences immune cell phenotype and changes systemic inflammatory mediators [21, 22].

Previous studies have used LPS stimulus to induce changes in NF-kappa B activation and Toll-like receptor 4 signalling pathways, simulating the exaggerated host response to infection through the release of adhesion molecules and cytokines [23]. When cells from patients with T2DM were exposed to LPS *in vitro*, the data were more heterogeneous, with studies finding increased, unchanged, or even decreased IL-1*β*, TNF, and IL-6 responses [24, 25]. After the training of our study, in LPS stimuli, neutrophil TNF-*α* and IL-8 production was higher and neutrophil IL-6 production was lower than the pretraining period. The imbalance in the production of cytokines in neutrophils from people with T2DM may be related to many factors including, for example, the rise in intracellular Ca^+2^ of the neutrophils caused by hyperglycaemia and the presence of advanced glycation end products [26].

Limitations of the study must be considered. The sample size was small and all participants were women. The inflammatory response to LPS is influenced by sex [27], and our results should be applied to males with caution. These limitations notwithstanding the lack of control in cytokine production by neutrophils in people with T2DM may result in inadequate activation of neutrophils and may influence the progress of diabetic complications, such as retinopathy, neuropathy, vasculopathy, and diabetic nephropathy [3, 28].

After dance training, participants with T2DM exhibited fewer neutrophils with fragmented DNA, suggesting that these cells were better protected against apoptosis. This is an important finding since neutrophils from patients with T2DM usually demonstrate defects in LPS-induced apoptosis [29]. Dysregulated neutrophil apoptosis contributes to pancreatic b-cell destruction and increases the number of proinflammatory cells and the onset of autoimmune or inflammatory diseases [30]. Furthermore, the functional longevity of neutrophils and neutrophil clearance from infectious sites are decreased with dysregulated apoptosis, which may contribute to the increased susceptibility to infection and severity of infection observed in people with diabetes [31].

Apoptosis is associated with the production of inflammatory markers. For instance, low levels of TNF-*α* reduce apoptosis in neutrophils. However, at high concentrations, TNF-*α* reverses the protective effects of granulocyte-macrophage colony-stimulating factor and interferon-*γ* [32]. Thus, the mechanisms underlying diabetes-induced cell death appear to involve the prolonged production of proinflammatory markers, among others [3]. This led us to hypothesize that the reduction in plasma inflammatory markers and the increase in anti-inflammatory cytokines after dance training observed in this study are causally related to decreased neutrophil apoptosis and the increased capacity of neutrophils to produce cytokines with LPS stimulation. Although further research is required to test this hypothesis, these events could increase protection against the advance of diabetic complications.

## 5. Conclusion

This study found reduced cell death in neutrophils from patients with T2DM and improved neutrophil response in neutrophils after 4 months of dance training. Moreover, even in the absence of a change in body composition, these changes were followed by increased plasma levels of anti-inflammatory IL-1ra and decreased plasma levels of inflammatory TNF-*α* and CRP. These findings may represent a useful tool to design nonpharmacological strategies to reduce inflammation and improve neutrophil clearance in patients with T2DM.

## Figures and Tables

**Table 1:** Baseline characteristics in the clinical and metabolic profiles of the participants.

	Control group	T2DM group
Physical and clinical aspects		
Age (years)	62.9 ± 1.8	70 ± 3^∗^
Height (m)	1.57 ± 0.01	1.6 ± 0.02
Diagnosis of DMT2 (years)	—	19.7 ± 5.5
Smokers (*n*)	2	0
Pedometer (steps/day)	4668 ± 973	7947 ± 2029
IPAQ—inactive (%)	13.3	0
IPAQ—minimally active (%)	33.4	25
IPAQ—active (%)	53.3	75
Systolic BP (mmHg)	117.1 ± 4.3	132 ± 3.6^∗^
Diastolic BP (mmHg)	73.9 ± 2.6	84.9±2.2^∗∗^
Plasma lipoprotein levels		
Cholesterol (mmol/L)	192.1 ± 7.4	166.3 ± 13
HDL (mmol/L)	57.9 ± 3.2	55.7 ± 5.1
LDL (mmol/L)	116.2 ± 7.4	92.6 ± 9.2
TG (mmol/L)	106 ± 9.2	90 ± 6

Note. The values are presented as the mean ± SE. ^∗^*p* < 0.05 control vs. T2DM; ^∗∗^*p* < 0.01 control vs. T2DM. Data are represented as the mean ± SE of 10-12 participants per group. mmHg: millimeters of mercury.

**Table 2:** Anthropometric values and plasma glucose, fructosamine, leptin, and FFA levels in participants with T2DM and healthy participants before and four months after dance training.

Anthropometric values, glycaemic control, analysis of leptin, and FFA	Precontrol	Postcontrol	Pre-T2DM	Post-T2DM
Total body mass (kg)	65.6 ± 3.2	64 ± 3.2	69.6 ± 4	68.8 ± 4
BMI (kg/m^2^)	26.6 ± 1.2	25.9 ± 1.2	28.4 ± 1.4	28.2 ± 1.5
Body fat (%)	35 ± 1.2	34.1 ± 1.3	36.5 ± 1.9	35 ± 2.4
Lean mass (kg)	40.5 ± 2.1	40.6 ± 2	44 ± 2	44.7 ± 2
Waist-hip ratio (cm)	0.87 ± 0	0.86 ± 0	0.92 ± 0	0.91 ± 0
Blood glucose (mmol/L)	104.5 ± 2.2	101.5 ± 2.9	141.9 ± 13.8^∗^	125.9±10.2^∗∗^
Fructosamine (*μ*mol/L)	252.5 ± 15.8	247.2 ± 35.7	277.3 ± 20.3	276.4 ± 32.1
Leptin (pg/mL)	156.3 ± 23.1	152.9 ± 28.1	117.8 ± 28.1	136.8 ± 19.7
FFAs (mEq/L)	0.2 ± 0	0.2 ± 0	0.2 ± 0	0.1 ± 0

Note. Data are represented as the mean ± SE of at least 7 participants per group. The values are expressed as the mean ± SE. ^∗^*p* < 0.0005 precontrol group vs. pre-T2DM group, ^∗∗^*p* < 0.01 postcontrol group vs. post-T2DM group. kg: kilogram; mmol/L: millimoles per litre; *μ*mol/L: micromole per litre; mEq/L: milliequivalent per litre.

## Data Availability

The data used to support the findings of this study are available from the corresponding author upon request.

## Supplementary Materials

Supplementary Materials Table A: drug classification used for characterization and matching in the study.
