# Supplementary material for: Dance Training Improves Cytokine Secretion and Viability of Neutrophils in Diabetic Patients
Source: Mediators Inflamm. 2019 Nov 20;2019:2924818. doi: 10.1155/2019/2924818 (PMC6886327; doi:10.1155/2019/2924818)
Supplement: Supplementary Materials — Table A: drug classification used for characterization and matching in the study. [file 2924818.f1.pdf]

Supplementary material

Table A: Drug classification used for characterization and matching in the study.

	Control group	T2DM group
**Medication**		
Participants without medication *(n)*	10	1
Insulin-dependent *(n)*	0	6
Insulin frequency *(days/week)*	0	7
Lipid-lowering agents *(n)*	4	1
Metformin family *(n)*	0	6
ACE inhibitor *(n)*	1	2
Beta-blocker I *(n)*	3	3
Diuretics *(n)*	1	1
Angiotensin II *(n)*	3	3

~~Note:~~ (1) Participants were paired in the ratios 1/1 and 1/2; (2) there was no pairing for lipid-lowering agents [reason: biochemical control was carried out by lipid analysis – Table 2]; (3) Data are represented as the mean ± SE of at least 10 participants per group. Abbreviations: angiotensin-converting enzyme, ACE.
